# Origin of Mangetotransport Properties in APCVD Deposited Tin Oxide Thin Films

**DOI:** 10.3390/ma13225182

**Published:** 2020-11-17

**Authors:** Krunoslav Juraić, Davor Gracin, Matija Čulo, Željko Rapljenović, Jasper Rikkert Plaisier, Aden Hodzic, Zdravko Siketić, Luka Pavić, Mario Bohač

**Affiliations:** 1 Ruđer Bošković Institute, Bijenička cesta 54, 10000 Zagreb, Croatia; davor.gracin@irb.hr (D.G.); zdravko.siketic@irb.hr (Z.S.); lpavic@irb.hr (L.P.); mario.bohac@irb.hr (M.B.); 2 Institute of Physics, Bijenička cesta 46, 10000 Zagreb, Croatia; mculo@ifs.hr (M.Č.); zrapljenovic@ifs.hr (Ž.R.); 3 High Field Magnet Laboratory (HFML-EMFL), Institute for Molecules and Materials, Radboud University, Toernooiveld7, 6525 ED Nijmegen, The Netherlands; 4 Elettra–Sincrotrone Trieste S.C.p.A., SS 14, km 163.5, 34149 Basovizza, Italy; jasper.plaisier@elettra.eu; 5 Central European Research Infrastructure Consortium, Strada Statale 14, km 163.5, 34149 Basovizza, Italy; aden.hodzic@ceric-eric.eu

**Keywords:** tin oxide, thin films, atmospheric pressure chemical vapour deposition transport properties, magnetoresistance, impedance spectroscopy, charge carrier mobility

## Abstract

Transparent conducting oxides (TCO) with high electrical conductivity and at the same time high transparency in the visible spectrum are an important class of materials widely used in many devices requiring a transparent contact such as light-emitting diodes, solar cells and display screens. Since the improvement of electrical conductivity usually leads to degradation of optical transparency, a fine-tuning sample preparation process and a better understanding of the correlation between structural and transport properties is necessary for optimizing the properties of TCO for use in such devices. Here we report a structural and magnetotransport study of tin oxide (SnO_2_), a well-known and commonly used TCO, prepared by a simple and relatively cheap Atmospheric Pressure Chemical Vapour Deposition (APCVD) method in the form of thin films deposited on soda-lime glass substrates. The thin films were deposited at two different temperatures (which were previously found to be close to optimum for our setup), 590 °C and 610 °C, and with (doped) or without (undoped) the addition of fluorine dopants. Scanning Electron Microscopy (SEM) and Grazing Incidence X-ray Diffraction (GIXRD) revealed the presence of inhomogeneity in the samples, on a bigger scale in form of grains (80–200 nm), and on a smaller scale in form of crystallites (10–25 nm). Charge carrier density and mobility extracted from DC resistivity and Hall effect measurements were in the ranges 1–3 × 10^20^ cm^−3^ and 10–20 cm^2^/Vs, which are typical values for SnO_2_ films, and show a negligible temperature dependence from room temperature down to −269 °C. Such behaviour is ascribed to grain boundary scattering, with the interior of the grains degenerately doped (i.e., the Fermi level is situated well above the conduction band minimum) and with negligible electrostatic barriers at the grain boundaries (due to high dopant concentration). The observed difference for factor 2 in mobility among the thin-film SnO_2_ samples most likely arises due to the difference in the preferred orientation of crystallites (texture coefficient).

## 1. Introduction

Transparent conductive oxides (TCO) are binary or ternary compounds containing one or two metallic elements. A very good balance of optical and electrical properties characterizes TCO materials. Widely used TCO include oxides such as ZnO, SnO_2_, In_2_O_3_ doped with metallic elements: Al-doped ZnO (AZO), Sn-doped In_2_O_3_ (ITO) and F-doped SnO_2_ (FTO) [1,2,3].

Among others, tin oxide (SnO_2_), also known as stannic oxide, is an n-type semiconductor (due to oxygen vacancies) with high optical transparency in visible spectral range (>85%) and a wide energy band gap (3.6 eV). It can be found in nature as a mineral known as cassiterite, and it is the main ore of tin [4]. It has a rutile-like crystal structure. The SnO_2_ thin films are chemically inert, scratch resistant, and can withstand high temperatures [5].

There are various chemical and physical methods for preparation of pure and doped SnO_2_ thin films: chemical vapour deposition, sol gel, spray pyrolysis, electron beam evaporation, vapour deposition, pulsed laser deposition, molecular beam epitaxy, thermal evaporation, reactive evaporation and magnetron sputtering, reactive magnetron sputtering, ion beam deposition [5].

Atmospheric Pressure Chemical Vapour Deposition (APCVD) is a process that enables deposition of vapour species in the form of thin solid films via suitable chemical reactions at atmospheric pressure. By careful choice of the deposition parameters the film properties can be systematically targeted. APCVD is often used in industry for thin film coatings deposition because of reduced costs due to low material consumption, high deposition rates and running costs (compared to low pressure systems) [6].

SnO_2_ thin films (pure and doped) have various applications in devices used in daily life: solar energy conversion, flat panel displays, electro-chromic devices, invisible security circuits, LEDs, transparent electrical conductors and non-colouring electrodes, in smart windows, for energy and illumination control, in anti-dazzling rear view windows, and non-emissive displays, low-emittance coatings for energy efficient windows, anti-frost coatings on car windows and transparent electrode for solar cells. In these applications, the film thickness normally lies in the 100–1000 nm range [5].

For photovoltaic application (as transparent front electrode), it is important that SnO_2_ is transparent for UV-VIS light while at the same time having very good conductivity. Electrical conductivity can be improved by increasing charge carrier density (doping with foreign atoms) or charge carrier mobility. The most favourable dopants are antimony (SnO_2_:Sb) and fluorine (SnO_2_:F). Fluorine-doped tin oxide (SnO_2_:F, FTO) exhibits good visible transparency owing to its wide band gap, while retaining a low electrical resistivity due to the high carrier concentration caused by the oxygen vacancies and the fluorine dopant. Highers numbers of charge carriers cause lower film transparency. Therefore, better conductivity should be achieved by optimizing charge carrier mobility.

Several different approaches have been reported with respect to how to improve charge carrier mobility of SnO_2_, including post-deposition heat treatment, deposition technique, substrate, doping control [7]. For example, charge carrier mobility can be improved by use of highly oriented substrate, use of tin tetrachloride as Sn precursor, higher methanol content.

In our previous publication [8], we reported in detail on the structural properties, examined by XRD, of undoped and doped SnO_2_ thin film samples deposited by APCVD with a short discussion about the influence on average transmittance in VIS part of the spectrum and specific surface resistivity at room temperature. In this work, we investigate in more detail the magnetotransport properties of such APCVD deposited SnO_2_ thin films in a wide temperature range and relate them with the structural properties. Transport properties were examined in a wide temperature range by impedance spectroscopy, DC resistivity, Hall effect and magnetoresistance. Structure and composition of SnO_2_ thin films were examined by Scanning Electron Microscopy (SEM), Grazing Incidence X-Ray Diffraction (GIXRD) and Time-Of-Flight Elastic Recoil Detection Analysis (TOF-ERDA). The analysis of the obtained experimental data shows that the scattering at grain boundaries is the dominant scattering process in this type of SnO_2_ samples and the small variation in the charge carrier mobility between the samples most likely stems from the difference in preferred orientation of crystallites (texture coefficient).

## 2. Materials and Methods

### 2.1. Thin Film Deposition by APCVD

SnO_2_ thin film samples were prepared by the APCVD on soda-lime glass substrates, in an industrial moving belt reactor [9] with constant-temperature zones. The reactor oven is interrupted with two slots perpendicular to the moving direction of the belt, supplied with a setup with a nozzle that enables the vapour or reactants to flow onto the heated glass surface. The reacting gas mixture was SnCl_4_, H_2_O, methanol and oxygen for undoped films, while for doped films, methanol was omitted and the freon gas was added into the vapour mixture. The vapour of reactants was produced in a “bubbler” by passing a carrier gas through the precursors at room temperature (methanol), or moderately heated to 50 °C (SnCl_4_) or 40 °C (H_2_O). The carrier gas for SnCl_4_, ethanol and H_2_O was nitrogen. The temperature of the glass substrate before and after deposition was 590 °C for samples S-590 and B-590, and 610 °C for samples S-610 and B-610 (see Table 1). The glass substrates were loaded by a belt into the furnace for SnO_2_ layer deposition. The single-layer deposition duration of 1.5 min and the post-deposition thermal treatment of some 30 min were adjusted by the belt speed. SnO_2_ film is formed by a very fast reaction of tetrachloride with water vapour in which methanol is often added as a moderator. For that reason, in the doped samples where the methanol was omitted, the growth rate was about 30% faster, which is similar to the results reported in Ref. [10].

As a result, two types of films were prepared. The first type (S-590 and S-610) was deposited in a one-step process, and the produced samples were intrinsic. The second type (B-590 and B-610) was prepared by depositing the first layer on the glass substrate in the same way as for the first type, while the second (top) layer, which was formed from the solution without methanol and with the addition of fluorine atoms in the form of freon (Chlorofluorocarbon, CFC), was deposited on the already-formed first layer.

### 2.2. Structural Characterization

#### 2.2.1. Scanning Electron Microscopy (SEM)

Sample surface morphology was analysed using JEOL, JSM 7000F field emission scanning electron microscope (FE-SEM, Zagreb, Croatia). The image acquisition conditions used were: 5 kV, 10 mm working distance, magnification 25k.

#### 2.2.2. Grazing Incidence X-Ray Diffraction (GIXRD)

As-deposited films were thoroughly studied by GIXRD, using the synchrotron radiation source. GIXRD was carried out at the MCX beamline [11] (Elettra synchrotron, Trieste, Italy) with a wavelength of the incident beam of 1.5498 Å (8 keV). The angle of incidence was set to 2.0° (a value much higher than the critical angle for total external reflection for SnO_2_ $\alpha_{c}=\sqrt{2\delta }\approx 0.37{}^{\circ}$, calculated from the SnO_2_ index of refraction, real part δ [12]). For the critical angle, the beam penetrates 10–20 nm below the surface and gives information about the surface morphology. For the angle of incidence α_i_ = 2.0° the beam penetrates much deeper and the GIXRD pattern contains the morphological information for the entire SnO_2_ layer.

#### 2.2.3. Time-of-Flight Elastic Recoil Detection Analysis (TOF-ERDA)

Atomic content and depth profiles of the elements in the samples were determined using TOF-ERDA. TOF-ERDA measurements were done by 20 MeV ^127^I^6+^ ions with 20° incidence angle toward the sample surface, and TOF-ERDA spectrometer positioned at the angle of 37.5° toward the beam direction. More details about TOF-ERDA setup used in this work can be found in Ref. [13].

### 2.3. Transport Characterization

#### 2.3.1. Impedance Spectroscopy

Sheet conductivity of SnO_2_ thin film samples was measured by impedance spectroscopy (Novocontrol Alpha-N dielectric analyser, Zagreb, Croatia) in the frequency range from 0.01 Hz to 1 MHz, in three subsequent temperature cycles: (i) cooling from 20 °C to −100 °C, (ii) heating from −100 °C to 220 °C, (iii) cooling from 220 °C to 20 °C. The temperature step for all cycles was 40 °C. Sheet conductivity data were normalized to the film thickness (Table 1) to obtain specific conductivity (S/cm).

#### 2.3.2. Magnetotransport

DC resistivity ρ, magnetoresistance and Hall effect measurements were done in the temperature interval from -270 to 27 °C and in magnetic fields up to 5 T. Thin film samples were cut for the Hall-bar geometry with typical dimensions (10 mm × 2 mm × 500 nm). Two current contacts and three pairs of Hall contacts were made by applying silver paint directly to the surface of the films. Magnetoresistance and Hall effect were measured simultaneously at fixed temperatures and magnetic field, B, going from −5 to 5 Tesla. Magnetic field was oriented perpendicular to the current through the sample and perpendicular to the surface of the films. Magnetoresistance data were symmetrized, $V_{\mathrm{xx}}=\frac{V_{\mathrm{xx}}\left(+B\right)+V_{\mathrm{xx}}\left(-B\right)}{2}$ (V_xx_ is the measured voltage), in order to eliminate the possible mixing of the Hall component and the Hall signal was antisymmetrized, $V_{\mathrm{yx}}=\frac{V_{\mathrm{yx}}\left(+B\right)-V_{\mathrm{yx}}\left(-B\right)}{2}$, in order to eliminate the possible mixing of the magnetoresistance component. Hall resistance $R_{\mathrm{yx}}=V_{\mathrm{yx}}/I$ was linear in magnetic field B throughout the whole temperature range for all samples, and the Hall coefficient was obtained as $R_{H}=\frac{V_{\mathrm{yx}}\;t}{I\;B}$, where I is the current and t the sample thickness. The magnetoresistance was determined as standard as $\frac{∆\rho }{\rho_{0}}=\frac{\rho \left(B\right)-\rho \left(0\right)}{\rho \left(0\right)}$.

## 3. Results and Discussion

### 3.1. Structural Properties

#### 3.1.1. Scanning Electron Microscopy

**Figure 1:**
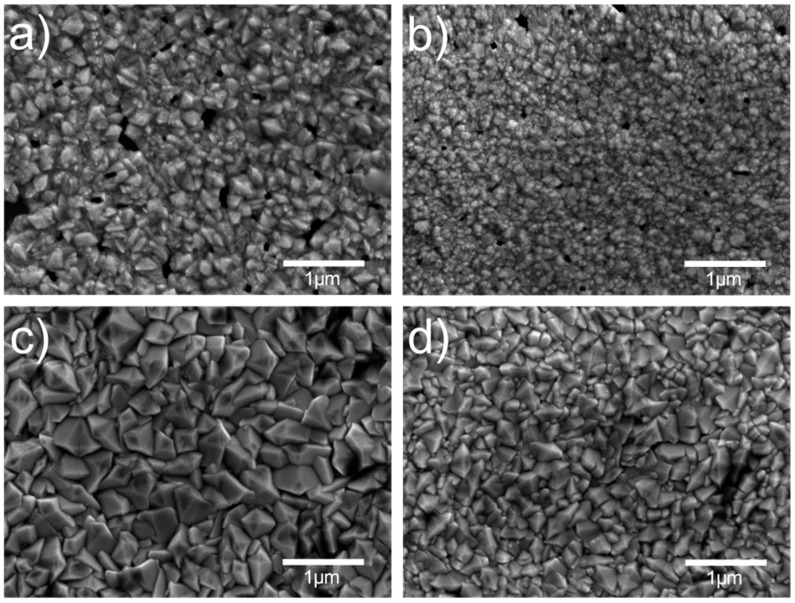
SEM images of SnO_2_ samples: (**a**) S-590, (**b**) S-610, (**c**) B-590 and (**d**) B-610. Average grain size calculated using Equation (1) are: 104 nm (S-590), 84 nm (S-610), 190 nm (B-590), 99 nm (B-610).

In Figure 1, SEM images of SnO_2_ thin film samples are presented. Average grain size was calculated from SEM image line profiles using the so-called “linear intercept technique” as follows [14]:

(1) $$\bar{D}=1.56\frac{C}{\mathrm{MN}}$$

where C is the total length of test line used, N is the number of intercepts and M is the SEM image magnification.

There is a significant difference in surface morphology (grain size and shape) for samples deposited in the one-step process—S-590 and S-610 (Figure 1a,b)—and samples deposited a in the two-step process—B-590 and B-610 (Figure 1c,d). Samples B-590 and B-610 have larger grains with sharp edges (pyramidal shape), while samples S-590 and S-610 have smaller grains with a more rounded shape. The samples deposited in the one-step process were deposited with addition of methanol, just as was done in the experiments reported in Refs. [10,15,16], which resulted in smoother films with smaller grains and a higher nucleation density, as reported in Ref. [15,16]. This effect was attributed to the removal of absorbed HCl, which is produced during deposition in reaction of SnCl_4_ with H_2_O. Removal of absorbed HCl leads to an increase in the number of adsorption sites for SnCl_4_ and H_2_O, and consequently to an increase in the micro-grain density and simultaneous decrease of grain size.

Higher deposition temperature produces samples with smaller grains (surface roughness) for both single-layer and bi-layer samples. This effect could be a result of competing gas-phase and/or surface reactions, which are a complex function of temperature and composition of reactants [17].

#### 3.1.2. Grazing Incidence X-Ray Diffraction

**Figure 2:**
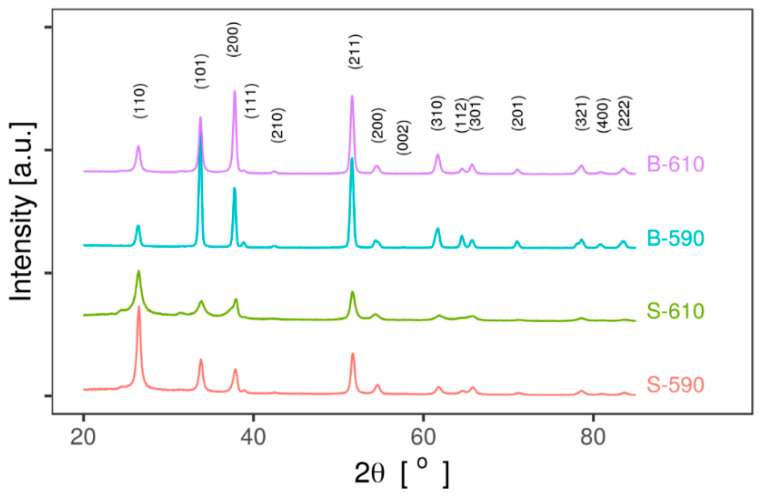
GIXRD diffractograms of SnO_2_ thin film samples. All visible peaks are indexed and labelled.

Figure 2 shows GIXRD patterns of the single-layer and bi-layer SnO_2_ thin film samples recorded at the fixed value of incident angle 2.0°. All observed reflections are unambiguously assigned to the tetragonal structure of SnO_2_ (space group *P*4_2_/*mnm*, SnO_2_ COD database ID: 9009082 [18]), mineralogical name cassiterite). XRD peaks (Figure 2) are fitted to PseudoVoight peak profiles.

Lattice parameters calculated from peak positions (labelled in Figure 2) are presented. Compared to values from the literature (a = 0.4737(3) nm and c = 0.3186(4) nm), all samples have larger values of the lattice parameter a, and equal values for the lattice parameter c. Variations of lattice parameters are due to the presence of intentional (fluor) and non-intentional (chlorine) dopant atoms with different ionic radii which substitute oxygen atoms in the lattice. 

There is also a significant variation in XRD peak intensity ratio from sample to sample, which is also different from the values expected based on the literature. This suggests the presence of preferred orientation (texture) in films. For quantitative analysis the texture coefficient for a selected XRD peak was calculated as intensity ratio of the selected XRD peak and the total sum of all fitted XRD peaks using the following formula [19,20]:

(2) $$\mathrm{TC}\left(h,k,l\right)=\frac{I\left(\mathrm{hkl}\right)/I_{0}\left(\mathrm{hkl}\right)}{\frac{1}{N}\sum_{1}^{N}I\left(\mathrm{hkl}\right)/I_{0}\left(\mathrm{hkl}\right)}$$

where I(h,k,l) is the measured relative intensity of the plane (h k l), I_0_(h k l) is the standard intensity of the plane (h k l) taken from literature (COD database, SnO_2_ COD ID: 9009082 [18]), and N is the number of reflections included in the calculations. The results for the four most intense XRD peaks are presented in Table 2. It is interesting that samples deposited at the lower temperature (S-590 and B-590) have significantly larger texture coefficients for (110), while B-590 and B-610 samples have larger (200) texture coefficients.

Average crystallite size was estimated using the standard Scherrer Equation [21]:

(3) $$D=\frac{K\lambda }{\beta_{\mathrm{hkl}}\cos\theta }$$

where β_hkl_ is XRD line width, D is crystallite size, K is shape factor (0.94) and λ (= 0.154 nm) is the wavelength of Cu K-α radiation. The mean values of average crystallite size obtained by Scherrer equation for the four most intense diffraction peaks are presented in Table 2. The much smaller values for crystallite size compared to grain size estimated from SEM images indicate that grains consist of several crystallites.

Samples deposited in the one-step process have smaller crystallite size calculated from GIXRD and grain size clearly seen from SEM images. Two possible effects/reasons could be responsible for smaller crystallite and grain size in the single-layer samples. First is the smaller thickness of single-layer samples, due to which it can be assumed that there are smaller crystallites and grains at the surface according to the standard thin film growth model [22]. The second one is variation in the deposition temperature. Samples deposited at the lower temperature have larger average crystallite size. Temperature affects a complicated set of chemical reaction mechanisms during the mixing of reactants in vapour phase and at the substrate surface.

#### 3.1.3. TOF-ERDA

**Figure 3:**
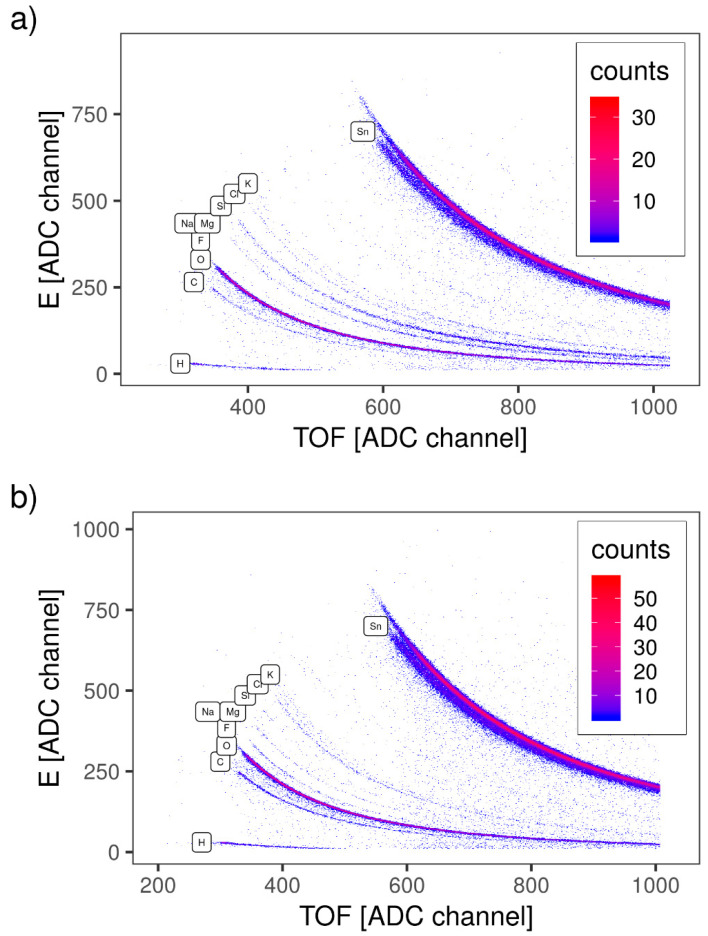
TOF-E map for SnO2 samples: (**a**) S-590 and (**b**) B-590. Traces of all detected elements are labelled.

**Figure 4:**
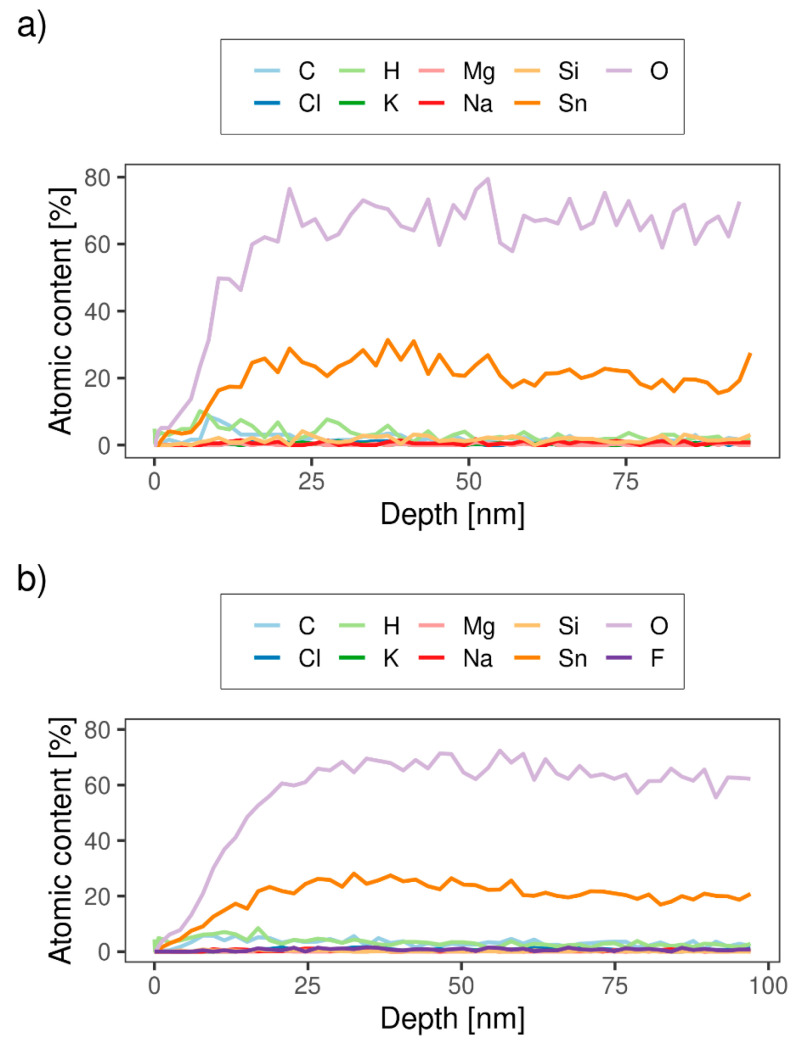
TOF-ERDA elemental depth profile calculated by Potku (slab analysis) for SnO_2_ samples: (**a**) S-590 and (**b**) B-590.

TOF-ERDA was employed for the elemental depth profiling of the S-590 and B-590 samples. Due to the overlapping of Sn and scattered I lines in TOF-E spectra (Figure 3a,b), only the first 10^18^ at./cm^2^ of sample depth was analysed. Energy spectra belonging to each element were analysed using simulation code Potku [23] (slab analysis) and the Monte Carlo (MC) code CORTEO [24]. Calculated depth profiles (Potku analysis, version 1.1) are presented at Figure 4a,b for S-590 and B-590 respectively. Since the slab analysis does not take into account detector energy resolution and all other contributions to the total energy spread (energy straggling and multiple scattering of incoming and recoiled ions), derived atomic concentrations were used only as input data for the MC simulation. Average atomic concentrations, calculated by MC simulations, are listed in Table 3. 

The results in Table 3 confirm that the sample deposited in the one-step process (S-590 and S-610) does not contain fluor, as is expected.

For both types of sample, the ratio of Sn and O atoms are stoichiometric (1:2), within the measured error, considering that part of O atoms are bonded to Si in substrate or C atoms at the sample surface. For samples deposited in the two-step process, the concentration of F atoms (dopant) is almost 1%, while for samples deposited in the one-step process, the amount of F atoms is below the detection limit (<0.1 at.%).

Small amounts of Si, K, Mg and Na, visible in TOF-E spectra (Figure 4a,b), could originate from the glass substrate, since the sample area is not fully covered by SnO_2_ film. Higher numbers of holes/cracks are expected for thinner samples (single-layer), which could explain the higher contribution of Si, K, Mg and Na in the sample S-590 (Table 3). 

### 3.2. Transport Properties

#### 3.2.1. Impedance Spectroscopy

**Figure 5:**
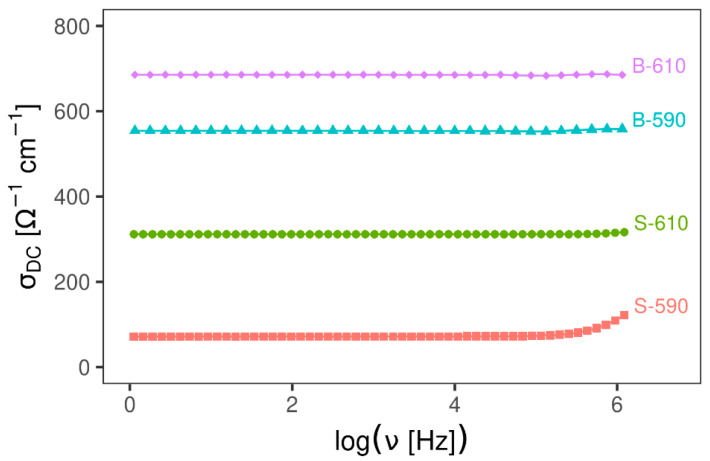
Electrical conductivity for SnO_2_ samples as a function of frequency at 20 °C.

Impedance spectroscopy results (Figure 5) show that all samples have a very high electrical conductivity, independent of frequency in a wide frequency range indicating fast electronic transport. As expected, samples deposited in the two-step process (B-590 and B-610) have higher conductivity compared to samples deposited in the one-step process at the same temperature because of doping. Only sample S-590 shows a very small dispersion at the highest frequency range. For samples with higher conductivity, a dispersion phenomena is also expected at higher frequencies that are above the limits of the experimental setup. In addition, electrode polarisation effects are not observed for any of the samples, indicating the absence of ion transport contribution to the electrical conductivity. 

#### 3.2.2. Magnetotransport Probe

**Figure 6:**
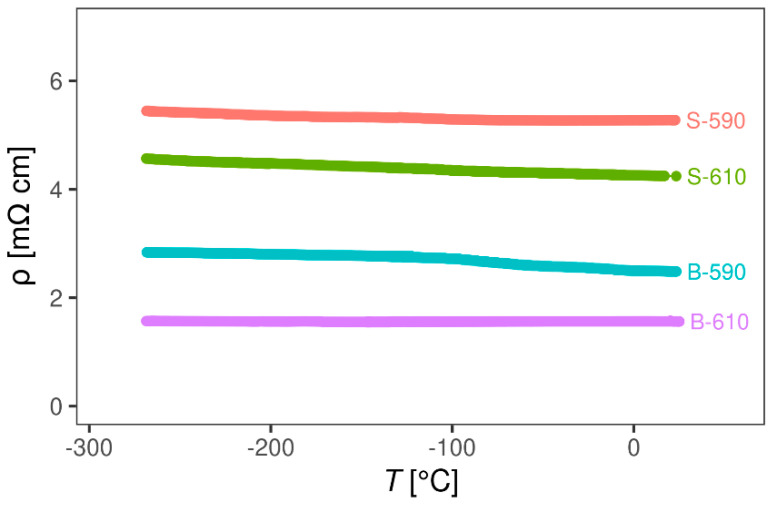
Resistivity of SnO_2_ samples as a function of temperature.

**Figure 7:**
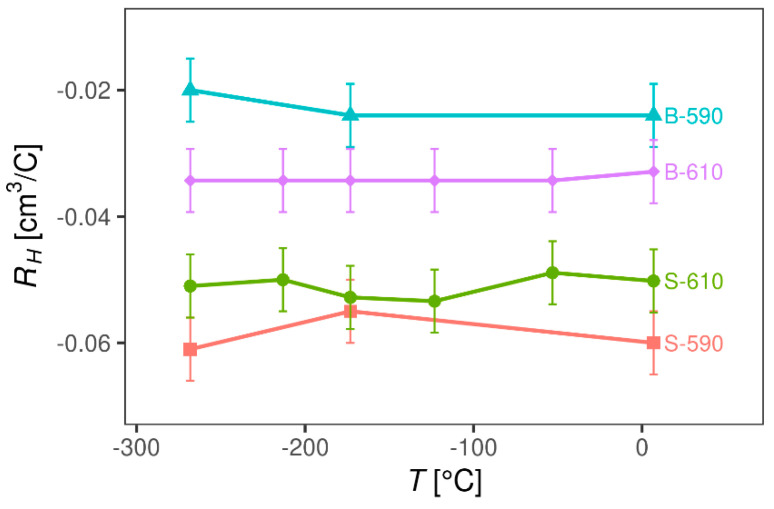
Hall coefficient R_H_ of SnO_2_ samples as a function of temperature.

Figure 6 shows the DC resistivity of the four SnO2 thin films as a function of temperature. Interestingly, for all samples, the DC resistivity has a negligible temperature dependence from room temperature down to −269 °C, indicating a metallic type of charge transport. The high temperature measurements confirmed that the DC resistivity stays almost independent of temperature up to 150 °C. Hall effect measurements showed that the Hall resistivity is linear in a magnetic field up to 5 T and that the carrier density extracted from the Hall coefficient *R*H is in the range 1–3∙× 10^20^ cm^−3^, which is a typical value for SnO2 films. As expected, *R*H is negative, indicating *n*-type free carriers and doped samples (B-590 and B-610) have a higher electron density (2–3 × 10^20^ cm^−3^) than the undoped ones (S-590 and S-610) (1 × 10^20^ cm^−3^). As can be seen in Figure 7, RH for all samples also shows a negligible temperature dependence, indicating that all samples behave as heavily doped semiconductors and that for both, doped and undoped samples, the Fermi level lies either in the conduction band or in the region where the conduction band is mixed with impurity levels. 

**Figure 8:**
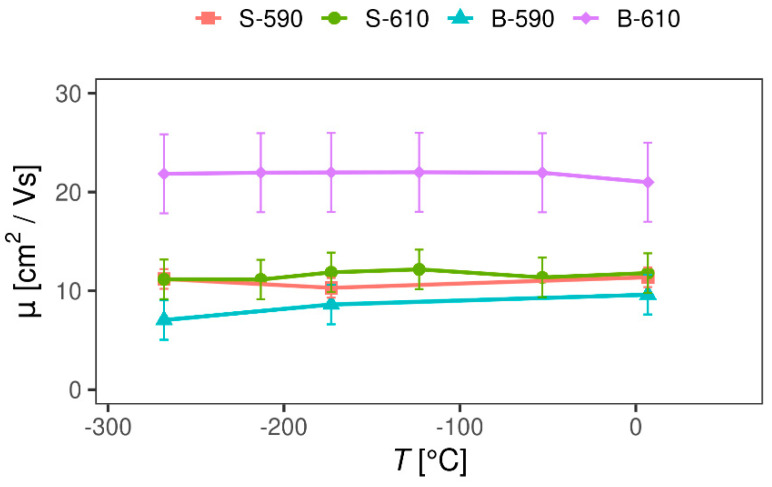
Charge carrier mobility of SnO_2_ samples as a function of temperature.

Having determined the DC resistivity *ρ* and the Hall coefficient *R*_H_, we are able to calculate the carrier mobility μ = R_H_/ρ, which is shown in Figure 8 for all four samples. Remarkably, charge carrier mobility is independent on temperature from room temperature down to −269 °C, in sharp contrast to behaviour found in conventional semiconductors, indicating the dominance of a non-trivial scattering mechanism.

There are several scattering mechanisms influencing the charge carrier mobility in doped semiconductors: electron–phonon scattering, scattering of electrons on ionized impurities, electron–electron scattering, scattering of electrons on neutral impurities, and inter-valley scattering [25]. The last three mechanisms are usually much less pronounced and can generally be ignored for a first approximation. Electron–phonon scattering is a standard scattering mechanism present in all materials and is especially pronounced at high temperatures. Scattering of charge carriers on ionized impurities is usually a second dominant scattering mechanism in doped semiconductors. This is due to the excitation of an electron from the impurity level to the conduction band (n-type) or excitation of an electron from the valence band to the impurity level (p-type) that leaves an uncompensated charge on the impurity. As mentioned earlier, our SnO2 samples are a heavily doped n-type material, intrinsically doped by the oxygen vacancies and extrinsically by the fluorine atoms, so that both electron–phonon and scattering on ionized impurities are expected to play a role in the charge transport. However, both scattering mechanisms show a pronounced temperature dependence [25], in sharp contrast to our negligible temperature dependence of charge carrier mobility (Figure 8), pointing towards the presence of an additional scattering mechanism with basically no temperature dependence.

The charge carrier mobility in polycrystalline samples is known to be determined by grain boundary scattering resulting from the electrostatic charge trapped at the intergrain boundaries, which sets up potential barriers to current flow, although such scattering usually also shows a pronounced temperature dependence. A theoretical model by Prins et al. [26] shows, however, that for certain parameter values the grain–boundary scattering can indeed produce a temperature-independent mobility. The model depends on bulk parameters—the carrier effective mass and the mean free path—and the grain boundary parameters—barrier height, barrier width, and a coefficient of sample inhomogeneity. (The barrier height is defined as the energy difference between the Fermi level and the top of the barrier and the transport in the interior of the grains is separated from the transport across the intergrain boundaries.) The model is tested on five Sb-doped SnO2 thin films with a different doping level. The films with a charge carrier density >10^18^ cm^−3^ showed temperature-independent carrier density (the interior of the grains is degenerately doped) and the sample with the highest dopant concentration showed a negligible temperature dependence of both the carrier density and the carrier mobility over a temperature range of nearly 300 °C, very similar to the behaviour found in our thin films. Moreover, the carrier mobility for the sample with the highest dopant concentration in Ref. [26] was found to be around 18 cm^2^/Vs which is very close to the values found in our samples (see Figure 8). Such behaviour is interpreted as originating from the fact that the Fermi level is situated well above the conduction band minimum at the grain boundaries and the negligible electrostatic barriers at the grain boundaries caused by the high dopant concentration (barrier height is negative). 

Having established that grain–boundary scattering is responsible for temperature-independent transport in our SnO2 thin films, let us now try to address the small difference in mobility found between the doped and undoped samples prepared at a different deposition temperature. By comparing the grain structure determined by SEM shown in Figure 1 with the charge carrier mobility determined by magnetotransport in Figure 8, no obvious correlation can be found. For example, in contrast to the expectations, the sample with the biggest grains B-590 turns out to have the smallest carrier mobility, while the biggest carrier mobility was found in the sample with the medium grains B-610. 

A relatively recent study by Wang et al. [27] indicated the importance of the preferred orientation of crystallites (texture coefficient) in limiting the charge carrier mobility in fluorine-doped thin SnO2 films prepared by APCVD. The main conclusion of this study is that the growth in texture coefficient (110) decreases, while the growth in the texture coefficient (200) increases the carrier mobility in SnO2 films. Looking at Table 2, we can see that the sample B-610, which has the biggest mobility, indeed has the biggest value of the texture coefficient (200), while the sample B-590 with the smallest mobility has the smallest value of the texture coefficient (200), in agreement with the results in Ref. [27]. We can say that the dominant scattering mechanisms in our SnO2 thin films are grain–boundary scattering, responsible for temperature independent carrier mobility, and crystallite scattering, possibly responsible for small differences in carrier mobility among the SnO2 samples prepared under slightly different conditions. More systematic study would be necessary in order to disentangle the contributions coming from the grain–boundary and crystallite–boundary scattering in our SnO2 samples and to establish a direct relationship to the carrier mobility.

**Figure 9:**
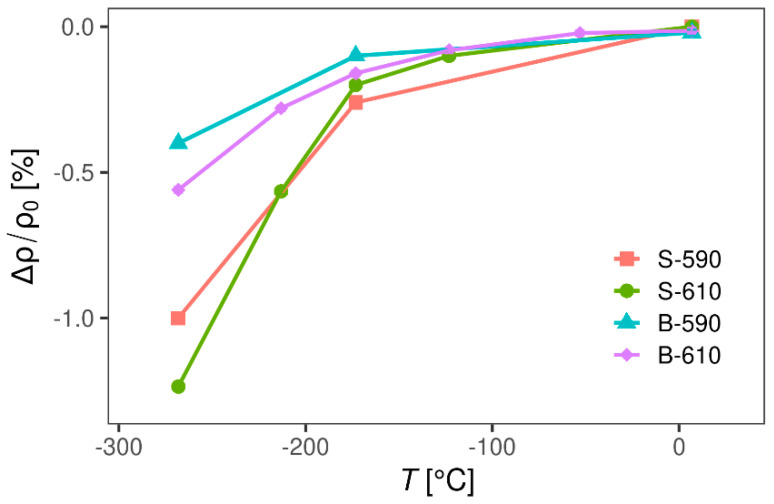
Magnetoresistance of SnO_2_ samples as function of temperature. Error bars are omitted in plot because they are order of experimental point symbol size.

Magnetoresistance (Figure 9) of all samples is small (<2%), negative and its value slowly increases with cooling. Negative magnetoresistance is rare in non-magnetic materials and usually has an exotic origin, indicating again that SnO_2_ samples do not follow simple metallic behaviour. However, negative magnetoresistance is found in impurity conduction of many semiconductors [28,29,30] and is often attributed to impurity band conduction [31,32], which is in accordance with the conclusions extracted from the temperature dependence of DC resistivity and the Hall coefficient. There are several theoretical models that try to resolve this behaviour; for example, weak localization [31], two band model with sharp mobility edge in the overlap region [32] and spin disorder [33]. The true nature of negative magnetoresistance in our samples is beyond the scope of this paper, but a more comprehensive study of this feature is likely to be part of future publications.

## 4. Conclusions

Undoped and fluorine-doped SnO_2_ thin films deposited by APCVD at two different temperatures, 590 °C and 610 °C, were studied by structural and magnetotransport probes. GIXRD revealed the polycrystalline nature of the thin films with the average crystallite size of 10–25 nm, while SEM indicated the presence of an additional inhomogeneity on a bigger scale in form of grains with the average size 80–200 nm. Samples deposited at 590 °C were found to have somewhat bigger grains, while samples deposited at 610 °C showed more (200) preferred orientation of crystallites. Hall effect measurements showed that the carrier density for both undoped and fluorine-doped samples was in the range 1–3 × 10^20^ cm^−3^ (assuming a substantial level of natural defects and unintentional doping) and has a negligible temperature dependence from room temperature down to −269 °C, indicating that the Fermi level lies either in the conduction band or in the region where the conduction band mixes with the impurity levels. Carrier mobility extracted from the Hall effect and DC resistivity turned out to have values 10–20 cm^2^/Vs and to be temperature independent down to −269 °C, showing that the usual scattering of phonons and ionized impurities play a minor role in these samples. Such temperature-independent transport properties are ascribed to the dominant grain boundary scattering, where the interior of the grains is degenerately doped, i.e., the Fermi level is positioned above the conduction band minimum, and due to high dopant concentration, the electrostatic barriers at the grain boundaries are negligible. However, for our samples, there was no obvious correlation between the grain size and the carrier mobility, as the sample with the biggest carrier mobility had medium size grains, while the sample with the smallest carrier mobility had the biggest grains. The highest charge carrier mobility was found in the sample with the largest (200) texture coefficient of crystallites, which is consistent with the results published in Ref. [27]. More systematic studies are needed to separate the influence of grain boundary scattering and scattering on the preferred orientation crystallites and to optimize the transport properties of SnO_2_ thin films prepared by APCVD.

## Figures and Tables

**Table 1:** Parameters for SnO_2_ thin film samples: sample labels, deposition process type, deposition temperature and layer thickness.

Sample Label	Deposition Process Type	Deposition Temperature (°C)	Thickness (nm)
S-590	one-step	590	390
S-610	one-step	610	300
B-590	two-step	590	920
B-610	two-step	610	710

**Table 2:** Results of GIXRD analysis for SnO_2_ samples: lattice parameters (a, c), texture coefficients for four most intense peaks (calculated using Equation (2)), and average crystallite size (calculated using Equation (3)).

Sample	Lattice Parameters		Preferred Orientation Texture Coefficient				Average Crystallite Size (nm)
	a (nm)	c (nm)	(110)	(101)	(200)	(211)	
S-590	0.474(9)	0.318(4)	0.144	0.090	0.104	0.151	17 ± 1
S-610	0.475(7)	0.318(8)	0.143	0.101	0.140	0.136	9 ± 3
B-590	0.475(9)	0.318(7)	0.019	0.141	0.102	0.166	22 ± 3
B-610	0.475(7)	0.318(5)	0.031	0.087	0.173	0.159	20 ± 3

**Table 3:** Summary of TOF-ERDA elemental analysis (MC simulation). Total atomic content is normalized to 100%.

Sample	H (at.%)	C (at.%)	O (at.%)	F (at.%)	Na (at.%)	Mg (at.%)	Si (at.%)	Cl (at.%)	K (at.%)	Sn (at.%)
S-590	2.8 ± 0.3	1.7 ± 0.2	62 ± 4	-	0.6 ± 0.1	0.18 ± 0.06	1.8 ± 0.2	0.7 ± 0.1	0.25 ± 0.06	29 ± 2
B-590	2.9 ± 0.3	3.2 ± 0.3	62 ± 4	0.9 ± 0.1	0.26 ± 0.06	0.03 ± 0.02	0.11 ± 0.03	0.9 ± 0.1	0.3 ± 0.1	29 ± 2
